# A nurse-centered edge–server wearable system for real-time monitoring of occupational stress in clinical care

**DOI:** 10.3389/fpubh.2026.1894346

**Published:** 2026-07-16

**Authors:** Lulu Fan, Hanyan Peng, Lang Shi, Yunhui Lu, Yuyang Song, Fei Peng, Changli Wan, Xiaoqiang Fan, Huilong Fan

**Affiliations:** 1 The Second Affiliated Hospital of Naval Medical University, Shanghai, China; 2 University of Electronic Science and Technology of China, Chengdu, Sichuan, China

**Keywords:** alert fatigue, digital health, edge computing, nurse occupational stress, patient safety, quality improvement, wearable monitoring, workforce wellbeing

## Abstract

**Background and objectives:**

Occupational stress among clinical nurses is a long-standing quality-of-care concern associated with burnout, attrition, lapses in patient safety, and erosion of workforce wellbeing. Continuous, nurse-centered monitoring of within-shift physiological stress states is therefore an emerging priority for nursing innovation and quality improvement (QI), yet routine clinical adoption of wearable monitoring is constrained by accuracy, response time, communication load, alert fatigue, and physiological differences across individual nurses. This study proposes the Nurse-centered Edge–Server Technology (NEST), a wearable edge–server framework for real-time occupational-stress monitoring designed to support QI-oriented screening and workflow awareness.

**Methods:**

Physiological and motion signals from wearable devices are processed locally with a compact feature set for rapid screening, while a richer feature set confirms suspected high-stress events through cascade alerting. A central server collects brief summaries from each device and returns small updates that personalize the model to each nurse over time. The framework was evaluated on the public Wearable Stress and Affect Detection (WESAD) dataset and on a continuous nurse-monitoring dataset collected in clinical wards, using subject-wise evaluation, out-of-fold supplementary event analysis, ablation experiments, and edge-equivalent runtime tests.

**Results:**

Lightweight screening preserved accuracy comparable to that of a richer feature set while reducing the feature dimension from 48D to 15D. In a held-out event-level test set of 24 positive events, the selected cascade preserved the 14/24 event hit count observed with Single48 while reducing false alerts from 9.68 to 1.88 per hour and total alerts from 10.65 to 2.10 per hour. Confidence intervals remained wide because the strict held-out stream protocol yielded 24 positive test events; supplementary all-subject and multi-split analyses were used as robustness checks for the primary held-out evaluation. In the edge-equivalent software setting, P95 latency remained in the millisecond range, the P95 CPU duty cycle was 6.77%, and 15D feature-summary upload was 4.69% of raw-stream upload. Chronological personalized updating increased sensitivity when feedback updates were available, but it did not improve overall F1 and lowered specificity; no-update controls showed no artificial gain.

**Conclusions:**

The retrospective evaluation supports the technical feasibility and alert-control value of a wearable edge–server pipeline that combines compact inference, cascade event confirmation, and personalized updating as an assistive QI-oriented monitoring approach. The study evaluates prediction behavior, alert burden, latency, and communication load; direct effects on burnout, nurse retention, workload reduction, patient-safety outcomes, or occupational-health improvement require prospective workflow studies. NEST is intended to augment clinical judgement and the relational role of nurses in occupational-health management and workforce sustainability.

## 1 Introduction

Nurse occupational stress is a long-standing concern at the intersection of patient safety, workforce wellbeing, and the sustainability of the nursing profession, and is therefore a natural focus for nursing innovation and quality improvement (QI). Nurses working in emergency departments, intensive-care units, and other high-acuity clinical settings are exposed, on a routine and prolonged basis, to occupational stress arising from workload peaks, irregular shifts, rapid task switching, and emotionally demanding patient interactions (1–4). Sustained exposure to these stressors has been repeatedly associated with burnout, absenteeism, and attrition among nursing staff, and is increasingly recognized as a contributor to reduced clinical vigilance and to lapses in patient safety (5, 6). These long-term occupational outcomes, however, should be distinguished from the short-term physiological stress states targeted by the present system. Nurse-centered Edge–Server Technology (NEST) is not designed to diagnose burnout or measure workforce outcomes directly; it aims to provide time-resolved screening of within-shift stress-related physiological and behavioral patterns that may inform QI-oriented awareness and supportive workflow review. Such within-shift states are shaped by episodes of emergency care, continuous observation, night duty, intensive patient communication, and rapid task transitions. Capturing this variation is essential for occupational-health management, for nurse-centered QI initiatives, and for timely supportive intervention, yet retrospective questionnaires and periodic self-reports—although informative for cross-sectional assessment—do not provide the continuous, time-resolved signal required to inform innovation in routine nursing practice.

Digital and technological innovation in nursing has rapidly expanded the toolkit available for continuous, in-workflow assessment of nurse wellbeing. Wearable devices and remote physiological-monitoring systems have begun to make long-duration acquisition of cardiovascular, autonomic, respiratory, and motion signals feasible during routine nursing work, and machine-learning-based mental-health monitoring has provided a methodological basis for stress detection and early warning that can be embedded within QI and implementation-science frameworks (7, 8). Heart rate, electrodermal activity, respiration, oxygen saturation, body temperature, and tri-axial acceleration carry observable information on autonomic response and task-associated behavior, and can in principle be acquired with limited interference in routine clinical workflows. Generic wearable stress-monitoring approaches nevertheless remain difficult to transfer directly into nursing workflows. Nurses move frequently, perform physical tasks, alternate between routine and high-acuity care, and may have limited opportunity to interact with an app or review dense alert streams during a shift. Physiological changes can also reflect movement, posture, protective-equipment discomfort, sleep deprivation, hydration, medication, caffeine intake, ordinary workload, and stress. Translating raw wearable streams into a clinically useful risk signal is therefore not reducible to acquiring more sensor data. The conversion must take place under three coupled constraints of wearable clinical deployment: edge-side compute, memory, and battery budgets that preclude continuous execution of high-capacity models; uplink bandwidth and patient-data confidentiality considerations that limit how much of the raw stream can leave the device; and clinical-workflow constraints that require a risk indication to be produced before the underlying physiological state loses operational relevance, and that demand alignment of the supporting model with each nurse's evolving physiological baseline (9–12).

Several lines of work have approached continuous physiological stress monitoring on constrained hardware. One line places computation near the sensor through edge or cloud–edge designs, reducing raw-stream transmission delay and limiting the exposure of physiological data, but the benefit holds only when feature compression preserves stress-discriminative content and when center-side coordination does not itself become a high-bandwidth upload process (9–12). A second line transfers high-capacity centralized stress-prediction architectures to wearable platforms, but the resulting compute, memory, and battery demand exceeds what smartwatches, patches, and similar low-power devices can sustain in continuous operation (13, 14); lightweight edge-only predictors mitigate this cost at the price of accuracy loss when physiological baseline, task context, or stress sensitivity differs across individual nurses. A third line analyses nurse occupational stress and nursing mental health through interpretable modeling—including job-stress network analysis, predictor-oriented modeling, and machine-learning identification of depression risk factors—and clarifies which factors carry predictive weight (15–17), yet remains essentially offline in nature and does not address adaptive, low-latency prediction on constrained hardware. Prior edge–center, privacy-aware, and federated or transfer-based studies further indicate that local response and model complexity can be partially decoupled (18–20), but usually treat communication efficiency, model updating, and prediction accuracy separately and rarely report event-level alert burden in continuous nurse-monitoring streams. A further consideration that is central to nursing quality improvement is that window-level classifiers, although often acceptable on segmented benchmark data, tend to generate dense alerts when applied to a continuous stream. In stress monitoring, false alerts differ from conventional physiological monitor alarms because they do not correspond to an immediately observable vital-sign emergency; repeated false stress alerts may therefore be ignored, produce unnecessary managerial attention, or increase perceived surveillance burden. Excessive alarms in clinical environments have been repeatedly identified as a workload-related risk that contributes to alert fatigue, alarm desensitization, and threats to patient safety in nursing practice (21). Taken together, these directions optimize individual axes—edge placement, model capacity, interpretability, or federated decoupling—but do not jointly handle model compactness, communication control, event-level alert behavior, and individual adaptation within a single continuous nurse-monitoring pipeline.

The present study addresses this gap by proposing the NEST, a nurse-centered wearable edge–server framework for real-time occupational-stress monitoring designed to support quality improvement in clinical care. NEST is a deployment-oriented monitoring pipeline that links three functions that are usually treated separately: compact edge screening, cascade confirmation for alert control, and nurse-specific updating from compact feedback. The innovation is not a new classifier alone, but a coordinated monitoring design for noisy, individualized, continuous nursing streams in which screening cost, false-alert burden, communication load, and adaptation are optimized together. This design directly follows from the workflow constraints identified above. Compact screening keeps routine inference feasible on the wearable side; 48D confirmation and temporal suppression reduce unnecessary stress alerts before escalation; and personalized updating addresses the fact that the same physiological fluctuation may have different meanings for different nurses or shifts. The retrospective evaluation examines this framework on the publicly available WESAD dataset and on a continuous nurse-monitoring dataset collected in clinical wards, against five deployment-relevant axes: window-level discrimination, event-level alerting, runtime cost, communication load, and drift adaptation.

Specifically, this paper makes three contributions. First, it formulates continuous nurse stress monitoring as a deployment-constrained task in which prediction must be timely, communication-bounded, alert-sparse, and adaptable to individual baselines rather than evaluated only as isolated window classification. Second, it implements this formulation as a 15D/48D cascade: a compact 15D representation supports low-cost screening, while a richer 48D representation and temporal-control rules confirm candidate events and suppress repeated false alerts caused by transient noise or task-related fluctuations. Third, it evaluates the resulting pipeline on WESAD and continuous nurse-monitoring streams, reporting not only window-level discrimination but also event recall, false alerts per hour, latency, communication load, module ablation, and chronological personalization behavior. The evidence supports the deployment value of compact, cascade-confirmed, and personalization-aware monitoring while keeping claims about direct occupational-health outcomes for future prospective studies.

## 2 Related work

Previous nurse stress prediction studies differ mainly in where computation is placed and how model updating is handled, and each setting carries different implications for nursing practice, workforce wellbeing, and quality improvement. Centralized models emphasize discrimination, whereas wearable-edge models emphasize response time and resource use. Personalized and collaborative methods further address subject variability and long-term model adaptation. The gap addressed by the present innovation lies between these settings: continuous, nurse-centered monitoring needs local decisions that are fast enough for use during shifts, alert outputs that remain manageable in long nursing workflows without triggering alert fatigue, and model updates that can track individual drift without frequent raw-data upload.

### 2.1 Enhancing accuracy with complex centralized models

Centralized stress-recognition and healthcare-stress monitoring studies commonly rely on engineered physiological features, server-side training, or deep architectures (22–29). Their advantage is access to richer temporal and multimodal structure, which can improve predictive performance (30). Under centralized settings, feature extraction, model training, calibration, and inference are not strongly limited by wearable-device resources. This allows models to use larger input representations and more complex decision boundaries than those usually feasible on low-power devices.

Deep neural-network stress detectors trained on WESAD and HRV-based multi-class stress classifiers show that richer non-linear representations can improve stress-state discrimination (31, 32). Cross-dataset ensemble work further indicates that generalization across wearable stress datasets remains a major challenge when models are trained on small or protocol-specific datasets (33). Personalized and multitask designs add subject information or related affective outcomes (34, 35). These modeling strategies are relevant to stress prediction because physiological responses often have temporal structure and subject-dependent variation. When computation and memory are sufficient, such methods can improve the capacity of the classifier to represent non-linear relationships among physiological, behavioral, and contextual variables. They also provide useful references for evaluating whether a lightweight method retains enough discriminative information after compression.

The same computational setting also limits clinical portability. Cloud and deep-learning pipelines may incur training and inference overhead beyond the budget of continuous wearable monitoring (36, 37), motivating lighter edge or cloud–edge alternatives (38, 39). In nurse monitoring, this limitation is not only a matter of speed. Continuous cloud-side processing may increase communication dependence, and high-dimensional feature transmission can raise privacy and bandwidth concerns. Therefore, centralized models are important baselines for discrimination, but they do not fully solve the deployment problem of low-latency nurse stress prediction.

### 2.2 Pursuing low latency via lightweight edge deployment

Wearable-edge studies reduce delay by placing compressed models or optimized inference routines directly on local devices (38, 40). Model compression, hardware-aware optimization, and power-aware inference lower computation and energy demand (41, 42). Compact mobile stress-detection models further support on-device recognition (43). These studies provide the basis for moving stress prediction closer to the sensing device and reducing dependence on centralized inference.

Low-latency deployment requires more than replacing a large classifier with a smaller one. The feature representation must also be compatible with wearable execution. A high-dimensional feature space may preserve more physiological and motion information, but it increases feature-construction cost, memory use, and upload traffic. A very compact representation reduces these costs, but it may remove stress-discriminative cues. This trade-off is especially important in nurse monitoring because physiological changes may be caused by stress, movement, workload intensity, or short-term sensor disturbance. The edge representation should therefore remain compact while retaining the physiological–motion information needed for local screening.

Inter-nurse variability remains a separate problem. Meta-learning for wearable physiological sensing (44), communication-efficient federated learning (45–47), federated nurse-scheduling research aimed at lowering fatigue levels (48), and adaptive mobile analytics (49–51) all point to the same constraint: an edge predictor must respond to context and baseline drift while remaining lightweight. In clinical monitoring, this limitation is amplified by alarm fatigue and burnout associated with excessive alerts (21). Dynamic nurse stress prediction therefore requires adaptation without losing edge-side efficiency.

### 2.3 Optimizing the edge-side accuracy-latency trade-off

Feature selection and communication control provide another route to the edge-side accuracy–latency trade-off. Prior studies have examined heart-rate-variability feature selection using random forests for mental stress quantification (52), sliding-window FFT with correlation analysis to reduce feature dimensions and computation (53), and event-triggered updates to reduce communication and extend battery life (54). Compact CNN–LSTM pipelines in related sensor-monitoring tasks show that lightweight architectures can remain competitive under constrained computation (55). These studies indicate that accuracy, latency, and communication cost can be jointly considered through feature design, model design, and update scheduling.

Compression alone is insufficient when the selected features lose stress-discriminative cues or the update rule cannot follow individual change. In nurse stress monitoring, the same physiological fluctuation may have different meanings under different action contexts. For example, a short-term change in heart activity or electrodermal response may reflect ordinary movement, temporary workload, or sustained psychological pressure. A compact edge representation should therefore preserve information that remains useful for distinguishing high-risk windows from normal task-induced fluctuations. This requirement motivates role-specific feature representations for screening, confirmation, and updating.

Communication and personalization must also be coordinated with the prediction process. A system that transmits every window or updates the local model too frequently may reduce latency gains obtained from edge inference. A system that never updates may become misaligned with a nurse's changing baseline. The method developed here therefore assigns compact screening to the edge, preserves cross-user information at the center, and invokes synchronization selectively. This design links low-latency local inference, communication-bounded updating, event-level alert control, and personalized correction in one continuous monitoring workflow.

## 3 Materials and methods

### 3.1 System model

The nurse stress monitoring system is modeled as an edge-center collaborative architecture composed of a set of monitored nurses, a set of wearable edge nodes, and a center-side coordination server. Let Equations 1–8 define the edge-center entities, local prediction, compact summaries, aggregation, and personalized correction used in the system model.


$$\begin{array}{l} N=\left\{n_{1},n_{2},\ldots ,n_{N}\right\} \end{array}$$ (1)

denote the set of nurses participating in the monitoring system, where *N* is the total number of users. Each nurse *n*_*i*_ is associated with a wearable sensing device that functions as a local edge node and continuously receives multimodal physiological and motion observations during nursing tasks.

At time *t*, the edge node corresponding to nurse *n*_*i*_ acquires a raw observation vector


$$\begin{array}{l} {\text{x}}_{i}\left(t\right)\in {\mathbb{R}}^{d}, \end{array}$$ (2)

where *d* denotes the dimensionality of the original sensor space. In the present setting, **x**_*i*_(*t*) may include heart rate, blood pressure, blood oxygen saturation, body temperature, electrodermal activity, respiration, and tri-axial acceleration, together with other context-relevant sensing channels when available. These raw streams are not directly used for centralized full-resolution inference, because such a strategy would impose excessive communication and computational burden under continuous monitoring conditions.

Instead, each edge node first applies an edge-side feature mapping function


$$\begin{array}{l} f_{e}:{\mathbb{R}}^{d}\to {\mathbb{R}}^{m},\;m\ll d, \end{array}$$ (3)

to obtain a compact local representation


$$\begin{array}{l} {\text{z}}_{i}\left(t\right)=f_{e}\left({\text{x}}_{i}\left(t\right)\right)\in {\mathbb{R}}^{m}. \end{array}$$ (4)

Here, *m* denotes the dimensionality of the distilled feature space. The role of this mapping is not merely dimensionality reduction. It provides a deployable interface between continuous multimodal sensing and low-latency local inference by retaining stress-relevant information in a compact representation.

Based on the distilled representation, the edge node performs real-time local prediction. Let


$$\begin{array}{l} {ŷ}_{i}\left(t\right)=M_{i}\left({\text{z}}_{i}\left(t\right)|\theta_{i}\left(t\right)\right) \end{array}$$ (5)

denote the local window-level prediction score, where *M*_*i*_(·) is the node-level predictor and θ_*i*_(*t*) denotes the current model parameters maintained at node *i*. In the implemented system, ŷ_*i*_(*t*) is treated as a calibrated high-stress risk score, which can be converted into a binary window-level decision through threshold selection for downstream cascade triggering and event aggregation.

The center node does not operate as a raw-data collection server. Instead, edge-center interaction is carried out through compact summaries, local prediction statistics, and lightweight parameter increments. Let


$$\begin{array}{l} {\text{s}}_{i}^{\left(r\right)}={\text{Ψ}}_{i}^{\left(r\right)}\left({\text{z}}_{i},{ŷ}_{i},\text{Δ}\theta_{i}\right) \end{array}$$ (6)

denote the summary uploaded by node *i* at update round *r*, where ${\text{Ψ}}_{i}^{\left(r\right)}\left(\cdot \right)$ represents the node-side summarization operator. The uploaded information may include distilled features, calibrated scores, local drift indicators, or compressed update signals, depending on the synchronization condition and current resource state.

After receiving summaries from multiple nodes, the center server updates the shared model prior through an aggregation operator


$$\begin{array}{l} \theta^{\left(r+1\right)}=G\left(\theta^{\left(r\right)},{\text{s}}_{1}^{\left(r\right)},{\text{s}}_{2}^{\left(r\right)},\ldots ,{\text{s}}_{N}^{\left(r\right)}\right), \end{array}$$ (7)

where θ^(*r*)^ denotes the shared model state at round *r*, and *G*(·) denotes the center-side aggregation function. The aggregation step updates cross-node knowledge from compact summaries without requiring continuous raw-stream upload.

The shared model is not pushed to each device as a complete replacement after every aggregation round. The center instead derives node-specific correction terms and sends lightweight updates to the edge. The local parameter evolution is written as


$$\begin{array}{l} \theta_{i}^{\left(r+1\right)}=\theta^{\left(r+1\right)}+\text{Δ}\theta_{i}^{\left(r\right)}, \end{array}$$ (8)

where $\text{Δ}\theta_{i}^{\left(r\right)}$ denotes the personalized correction term for node *i*. The center retains the population-level prior, and the edge node keeps the correction needed for individual physiological patterns and task-dependent drift.

The monitoring loop begins with windowing of incoming physiological and motion streams at each edge node. Window-level features are scored locally, and only selected summaries, score statistics, or update increments are transmitted. Center-side aggregation then produces lightweight correction terms for local adaptation. Time-critical scoring remains on the wearable side; slower population-level updating is handled by the center.

The mathematical model above describes the intended edge–center operating logic. In the empirical evaluation, this logic is instantiated by a calibrated random-forest cascade using fixed 15D and 48D feature representations, subject-wise train/test splits, threshold selection outside the held-out test subjects, event aggregation over continuous streams, edge-equivalent runtime testing, communication-load analysis, module ablation, and chronological personalization auditing. The privacy, dispatch, near-edge offloading, and full cross-node aggregation equations define system-design constraints and communication interfaces for prospective deployment rather than separately validated hospital functions in the present study.

The model follows three deployment assumptions. Edge–center communication is available but budgeted, so upload frequency and message size must be controlled through feature compression, summary transmission, and selective synchronization. Wearable devices are computationally constrained, requiring local processing to remain lightweight over long monitoring periods. Sensor noise, transient artifacts, and task-induced fluctuations are treated as expected input conditions in the monitoring stream.

This division assigns immediate risk scoring to the edge and lower-frequency knowledge integration to the center. The same separation structures the feature-distillation, incremental-updating, and collaborative-optimization modules described below.

### 3.2 Problem formulation

Given the real-time multimodal observation **x**_*i*_(*t*) collected from nurse *n*_*i*_, the goal of the edge node is to estimate the current window-level stress risk and determine whether the window should be treated as a high-risk candidate for downstream alerting The deployment-aware objective and its computational, communication, synchronization, and personalization constraints are summarized in Equations 9–15:


$$\begin{array}{l} y_{i}\left(t\right)\in \left\{0,1\right\}, \end{array}$$ (9)

where *y*_*i*_(*t*) = 1 denotes a high-risk window and *y*_*i*_(*t*) = 0 denotes a non-high-risk window. The target is not offline stress classification on fixed data; it is timely high-risk screening under limited edge computation, bounded communication, and low-frequency synchronization.

Accordingly, the nurse stress prediction task is formulated as a deployment-aware optimization problem. Let *f*_*e*_(·) denote the edge-side feature mapping, *M*_*i*_(·∣θ) denote the local predictor, *D*(·) denote end-to-end response delay, and Acc(·) denote predictive performance. The overall objective is defined as


$$\begin{array}{l} \underset{\theta ,f_{e}}{\min}F\left(\theta ,f_{e}\right)=D\left(f_{e}\left({\text{x}}_{i}\left(t\right)\right),M_{i}\left(\cdot |\theta \right)\right)-\lambda \text{ Acc}\left(\theta ,f_{e}\right), \end{array}$$ (10)

where λ>0 is a trade-off coefficient balancing timeliness and prediction quality.

Wearable-edge feasibility imposes four constraints. The feature-construction and local-inference delay must remain within the real-time budget:


$$\begin{array}{l} D\left(f_{e}\left({\text{x}}_{i}\left(t\right)\right),M_{i}\left(\cdot |\theta \right)\right)\leq 100\text{ms}. \end{array}$$ (11)

Second, the loss of predictive performance relative to the offline reference model should remain bounded:


$$\begin{array}{l} \text{Acc}\left(\theta ,f_{e}\right)\geq {\text{Acc}}_{\text{baseline}}-0.05. \end{array}$$ (12)

The 100 ms latency bound is used as an engineering budget for near-real-time risk display and feedback scheduling. The 0.05 performance-loss bound defines a practical tolerance for choosing a lightweight screening representation: the compact edge path should reduce latency, memory use, and communication load while remaining within five absolute percentage points of the higher-information offline reference under the same evaluation protocol. Third, the dimensionality of the transmitted edge representation must remain compact:


$$\begin{array}{l} m\leq m_{\max}, \end{array}$$ (13)

where *m* is the dimension of the distilled feature space and *m*_max_ is the maximum permitted communication dimension. Fourth, synchronization and model updating cannot be performed arbitrarily often, and the update interval must satisfy


$$\begin{array}{l} T\geq T_{\min}, \end{array}$$ (14)

where *T* denotes the update period and *T*_min_ is the minimum allowable interval under the target clinical workload. Finally, the system must preserve a shared global prior while allowing node-level personalization, which is written as


$$\begin{array}{l} \theta_{i}^{\left(r+1\right)}=\theta^{\left(r+1\right)}+\text{Δ}\theta_{i}^{\left(r\right)}, \end{array}$$ (15)

where θ^(*r*+1)^ denotes the updated center-side shared model and $\text{Δ}\theta_{i}^{\left(r\right)}$ denotes the node-specific correction term.

The problem is therefore a constrained streaming-prediction task. High-risk discrimination must be preserved while latency, communication, feature dimension, and update frequency remain bounded. The key design question is what to compute locally, what to transmit, and when to correct the local model after individual drift appears.

## 4 Proposed NEST pipeline

The method instantiates the system model as a streaming monitoring pipeline. Each window is converted into compact physiological–motion statistics, scored on the edge, and passed to cascade alerting or low-frequency center-side updating when needed. The pipeline is designed around bounded latency, limited uplink traffic, maintainable personalization, and stream-level alert behavior.

The following subsections specify the implemented components: local risk scoring, role-specific feature distillation, incremental personalization, and resource-coordinated edge–center operation. The notation from Section 3 is retained, but the focus shifts to executable model behavior.

### 4.1 Real-time stress prediction framework

Clinical monitoring requires a risk score before the window loses operational relevance. Section 3.2 defined this objective formally; the present subsection describes how edge screening, cascade confirmation, and center-side correction are connected during operation.

Three deployment conflicts determine the framework design. High-dimensional physiological and motion streams contain useful stress cues, but continuous processing of these streams is costly on low-power devices. Population-level training gives a stable starting model, yet it cannot represent every nurse's baseline, work rhythm, or stress sensitivity over time. Center-side learning needs cross-node information, but raw-stream upload is inefficient and unsuitable for privacy-sensitive monitoring.

These conflicts are handled by separating representation compression from model correction. Feature distillation supplies the compact input used for edge screening. Incremental learning modifies the local model when recent observations indicate baseline drift. The edge path remains lightweight, and the local predictor is not frozen after deployment.

**Figure 1:**
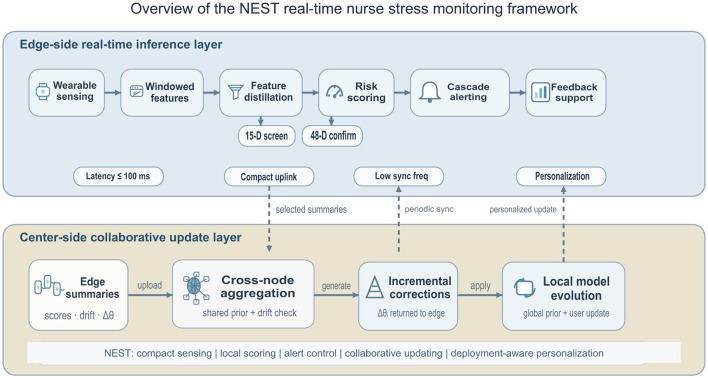
Overview of the proposed NEST framework for real-time nurse occupational-stress monitoring.

The pipeline has an edge inference layer and a center update layer. At the edge, streaming observations are segmented, converted into compact stress-related features, and scored by a calibrated local model. Candidate windows can then be passed to cascade confirmation. At the center, compact summaries and local update signals are aggregated at lower frequency. Returned updates take the form of local correction terms, not full-model replacements. The overall architecture and operating flow of NEST are shown in Figure 1.

The edge node handles time-sensitive scoring and alert support. Center-side computation is reserved for slower model maintenance and personalization. The resulting risk outputs are intended to inform workload buffering, task adjustment, or sustained-risk review, while final workflow decisions remain under clinical management rules.

Nurse stress prediction is treated here as a streaming, resource-constrained, and personalization-sensitive task. Compact edge inference provides the low-latency path, and center-side correction provides cross-user updating and individual adaptation.

### 4.2 Edge-side feature distillation mechanism

Efficient compression of high-dimensional multimodal signals is a prerequisite for real-time stress prediction on resource-constrained edge devices. In nurse monitoring scenarios, the raw input stream typically includes heterogeneous physiological and motion signals, such as heart rate, blood pressure, oxygen saturation, body temperature, electrodermal activity, respiration, and tri-axial acceleration. Although these signals jointly describe physiological status and task-related behavior, direct modeling of the full raw stream on the device side is impractical for sustained edge deployment. It increases feature extraction and inference latency, amplifies redundancy and sensor noise, and makes the prediction pipeline more sensitive to cross-modal heterogeneity. Conventional compression strategies, including fixed statistical summaries or generic dimensionality reduction, are computationally convenient, but they often fail to preserve the transient and context-dependent patterns that are most relevant to stress recognition, especially for non-stationary signals such as HRV and EDA. More importantly, such methods usually ignore the semantic interaction between physiological responses and nursing actions. More broadly, knowledge distillation has been explored in healthcare model compression as a way to preserve predictive utility in lighter deployable models (56).

**Figure 2:**
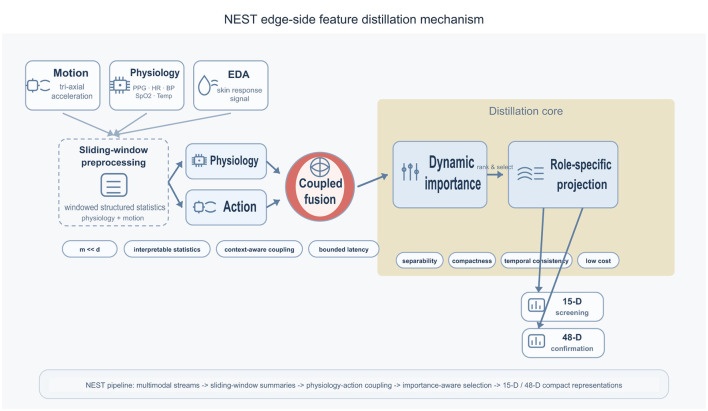
Schematic of the NEST edge-side feature distillation mechanism.

To address this limitation, we design an edge-side feature distillation mechanism that maps heterogeneous sensor streams into a compact and stress-sensitive subspace. In the implemented system, this mechanism is instantiated through interpretable structured statistics extracted on sliding windows, and the resulting compact representations are deployed in two role-specific forms: a 48-dimensional confirmation representation and a 15-dimensional lightweight screening representation. In abstract form, the feature construction, multimodal interaction, dynamic selection, compactness, and edge-side design criteria are formalized in Equations 16–25.


$$\begin{array}{l} {\text{z}}_{t}=f_{\phi }\left({\text{x}}_{t}\right),\;{\text{z}}_{t}\in {\mathbb{R}}^{m},\;m\ll d, \end{array}$$ (16)

where *f*_ϕ_(·) denotes a lightweight feature-construction operator for edge-side representation building. The mapping is constrained to retain stress-relevant structure while keeping edge-side computation and communication cost bounded. The edge-side feature-distillation process is summarized in Figure 2.

#### 4.2.1 Physiological–action coupled representation

Stress responses during nursing tasks depend on both physiological variation and action context. An elevated heart rate during routine movement may indicate workload, whereas a similar elevation during a high-intensity intervention may indicate stress accumulation. The distillation module therefore keeps physiological and motion signals coupled during feature construction.

We first decompose the raw observation into a physiological component **p**_*t*_ and an action component **a**_*t*_, and summarize them separately:


$$\begin{array}{l} {\text{h}}_{t}^{p}=\phi_{p}\left({\text{p}}_{t}\right),\;{\text{h}}_{t}^{a}=\phi_{a}\left({\text{a}}_{t}\right), \end{array}$$ (17)

where ϕ_*p*_(·) and ϕ_*a*_(·) denote lightweight feature-summary operators for physiological and motion-related information, respectively. In the implemented system, these summaries are derived from time-domain, frequency-domain, and motion-statistic features extracted on sliding windows. Simple concatenation cannot represent how action context changes the meaning of physiological fluctuation, so an interaction term is added to form the joint representation


$$\begin{array}{l} {\text{u}}_{t}=\left[{\text{h}}_{t}^{p},{\text{h}}_{t}^{a},\psi \left({\text{h}}_{t}^{p},{\text{h}}_{t}^{a}\right)\right], \end{array}$$ (18)

where ψ(·, ·) encodes the context-dependent relationship between current physiological variation and current nursing action.

Cross-modal association is represented with a feature interaction matrix **W**, whose entries quantify coupling between physiological and action dimensions. The interaction term is written in bilinear form as


$$\begin{array}{l} {\text{u}}_{t}=\left[{\text{h}}_{t}^{p},{\text{h}}_{t}^{a},{\left({\text{h}}_{t}^{p}\right)}^{⊤}\text{W}{\text{h}}_{t}^{a}\right]. \end{array}$$ (19)

This design allows motion signals to act as contextual modifiers for physiological responses. As a result, the feature construction stage can better distinguish normal action-induced fluctuations from stress-related abnormal responses before classification.

#### 4.2.2 Dynamic feature importance and low-dimensional subspace selection

After constructing the coupled representation, the next step is to retain only the most informative dimensions for edge-side inference. In nursing scenarios, however, the relevance of a feature is not static. During some periods, stress may be better reflected by short-term HRV instability; during others, the interaction between motion disturbance and physiological response may become more informative. A fixed projection matrix does not explicitly reflect the time-varying structure of stress-related signals.

To address this issue, we define a dynamic importance score for each candidate feature. For the *k*-th feature at time *t*, its importance is computed as


$$\begin{array}{l} s_{k}\left(t\right)=\alpha I_{k}^{\text{clin}}\left(t\right)+\beta I_{k}^{\text{stab}}\left(t\right)+\gamma I_{k}^{\text{ctx}}\left(t\right),\;\alpha +\beta +\gamma =1, \end{array}$$ (20)

where $I_{k}^{\text{clin}}\left(t\right)$ measures its clinical discriminative contribution, $I_{k}^{\text{stab}}\left(t\right)$ measures short-term statistical stability, and $I_{k}^{\text{ctx}}\left(t\right)$ measures sensitivity to the current action context. The score favors features that are discriminative, locally stable, and informative under the current task context; large variance alone is not sufficient. A retained feature should not only respond to stress, but should also remain locally stable enough for continuous monitoring and remain meaningful under the current task context.

Candidate dimensions are ranked by *s*_*k*_(*t*), and the retained indices define the edge-side stress-sensitive subspace. In the implementation, this selection is performed offline on training-derived data only to obtain two fixed representations: 48D for confirmation and 15D for lightweight screening. Held-out test subjects are not used to rank, select, or tune the retained feature dimensions. The 48D set is retained as the higher-information confirmation representation, whereas the 15D set is selected as a lower-cost screening representation from the same candidate feature table. The final feature names are reported in the study-design subsection to make the retained representations reproducible. Let $I_{m}$ denote the selected index set. The final distilled representation is


$$\begin{array}{l} {\text{z}}_{t}={\text{Π}}_{I_{m}}\left({\text{u}}_{t}\right), \end{array}$$ (21)

where $\Pi_{I_{m}}\left(\cdot \right)$ denotes projection onto the selected dimensions. The selected index set defines two fixed deployment feature sets: one for screening and one for confirmation.

#### 4.2.3 Multimodal fusion constraints and design criteria

The distilled representation must preserve class separation after compression and remain inexpensive enough for edge execution. We evaluate the compact feature space through separability, compactness, and computational feasibility.

Let **z**^(*c*)^ denote the distilled features associated with stress state *c*. We characterize inter-class separation and intra-class compactness as


$$\begin{array}{l} J_{\text{sep}}=\underset{c_{1}\neq c_{2}}{\sum }||𝔼\left[{\text{z}}^{\left(c_{1}\right)}\right]-𝔼\left[{\text{z}}^{\left(c_{2}\right)}\right]{||}_{2}^{2}, \end{array}$$ (22)

$$\begin{array}{l} J_{\text{compact}}=\underset{c}{\sum }𝔼\left[||{\text{z}}^{\left(c\right)}-𝔼\left[{\text{z}}^{\left(c\right)}\right]{||}_{2}^{2}\right]. \end{array}$$ (23)

The separation term keeps different stress states apart in the low-dimensional space, and the compactness term limits within-class dispersion caused by noise and irrelevant contextual variation.

In addition, because stress monitoring is performed over continuous sliding windows, the distilled representation should not fluctuate excessively due to transient sensor disturbance. We impose a temporal consistency regularizer:


$$\begin{array}{l} L_{\text{temp}}=\underset{t}{\sum }\omega_{t}||{\text{z}}_{t+\text{Δ}t}-{\text{z}}_{t}{||}_{2}^{2}, \end{array}$$ (24)

where ω_*t*_ is a task-dependent temporal weight. The term suppresses non-informative feature jumps without forcing genuine stress transitions to become overly smooth.

The edge-side design criterion is written as


$$\begin{array}{l} L_{\text{design}}=-J_{\text{sep}}+\lambda_{1}J_{\text{compact}}+\lambda_{2}L_{\text{temp}}+\lambda_{3}C\left(f_{\phi }\right), \end{array}$$ (25)

where *C*(*f*_ϕ_) denotes the computational cost of the feature mapping and λ_1_, λ_2_, λ_3_>0 are trade-off coefficients. Here, this expression serves as a design-oriented formulation for compact feature construction in the implemented system. In practice, both the 48D confirmation representation and the 15D screening representation are constructed from interpretable structured statistics selected offline, and both are paired with random-forest prediction, cross-fit estimation, sigmoid calibration, and threshold selection.

The edge-side distillation module produces a compact feature space that retains clinically relevant physiological–action cues while satisfying low-latency execution requirements. The selected 15D and 48D representations then provide the inputs for personalized learning and edge–center optimization.

### 4.3 Personalized incremental learning and edge-center collaborative evolution

**Figure 3:**
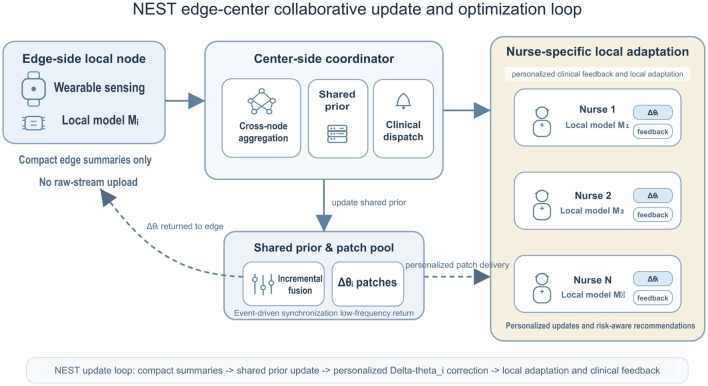
Schematic of the NEST edge–center collaborative update and personalized adaptation process.

The following subsection defines the intended personalization and edge–server update interface of NEST. In the present retrospective evaluation, this component is empirically evaluated through chronological feedback-based updating. Live multi-node hospital deployment and real-time server patch delivery remain deployment-stage extensions. The clinical-dispatch and personalized-feedback elements in Figure 3 therefore represent the planned deployment interface and workflow-support pathway.

Long-term nurse monitoring cannot rely on a model trained once and left unchanged. Shift schedule, workload, sleep state, and individual physiological baseline can alter the score distribution. A fixed decision boundary may become too conservative for one nurse and too sensitive for another, reducing both risk-score reliability and alert quality. Personalized incremental learning is used to update the edge predictor with recent individual evidence while retaining a center-side population prior.

The proposed mechanism separates global knowledge from local adaptation. The center maintains a shared initialization learned across users, whereas each edge node performs lightweight updates using recent local observations. Instead of transmitting raw multimodal streams, the edge uploads compact summaries, prediction statistics, and local parameter increments. The center then performs quality-aware aggregation and returns only compressed differential patches that are relevant to the current local drift state. The resulting update cycle consists of shared initialization, local adaptation, server aggregation, and compressed patch return. The shared initialization, local regularization, feature recalibration, compact patching, and synchronization trigger are described in Equations 26–35.

#### 4.3.1 Meta-initialized edge adaptation

Let θ_meta_ denote the shared initialization maintained by the center. This initialization is learned across multiple edge nodes so that the starting model remains broadly effective across individuals while avoiding excessive bias toward a small subset of users. To improve cross-user stability, the initialization is optimized by jointly considering the average task loss over nodes and the variation of that loss across nodes:


$$\begin{array}{l} \underset{\theta_{\text{meta}}}{\min}\frac{1}{N}\overset{N}{\underset{i=1}{\sum }}L_{i}\left(\theta_{\text{meta}}\right)+\lambda_{v}\text{Var}\left(L_{1},\ldots ,L_{N}\right), \end{array}$$ (26)

where *L*_*i*_(·) denotes the task loss on node *i*, and λ_*v*_ controls the stability penalty across users. This objective yields a global prior that is not only accurate on average but also less sensitive to inter-individual variability.

During deployment, node *i* initializes its local model from $\theta_{\text{meta}}^{\left(r\right)}$ at round *r* and performs a small-step update using recent local data $D_{i}^{\left(r\right)}$:


$$\begin{array}{l} \theta_{i}^{\left(r\right)}=\arg\underset{\theta }{\min}\left[L_{i}\left(\theta ;D_{i}^{\left(r\right)}\right)+R\left(\theta ,\theta_{\text{meta}}^{\left(r\right)}\right)\right], \end{array}$$ (27)

where the regularization term constrains the local model from drifting excessively far from the shared prior. A quadratic penalty is adopted in practice:


$$\begin{array}{l} R\left(\theta ,\theta_{\text{meta}}^{\left(r\right)}\right)=\lambda_{i}^{\left(r\right)}||\theta -\theta_{\text{meta}}^{\left(r\right)}{||}_{2}^{2}, \end{array}$$ (28)

where $\lambda_{i}^{\left(r\right)}$ is adjusted according to the current drift state. Local samples that remain close to the shared prior permit a weaker constraint; stronger drift increases the penalty to prevent fitting short-lived fluctuations. The update rule adapts to individual dynamics while preserving the shared discriminative structure.

#### 4.3.2 Dynamic feature recalibration for individual pattern reinforcement

Feature relevance also varies by nurse, so parameter updates alone are insufficient. HRV and electrodermal variation may dominate for one user, whereas motion-modulated physiological response may dominate for another. The edge node therefore recalibrates the distilled representation directly while preserving the feature-space definition.

Let **z**_*i, t*_ denote the distilled feature vector of node *i* at time *t*. The recalibrated representation is defined as


$$\begin{array}{l} {\tilde{\text{z}}}_{i,t}^{\left(r\right)}={\text{a}}_{i}^{\left(r\right)}⊙{\text{z}}_{i,t}, \end{array}$$ (29)

where ${\text{a}}_{i}^{\left(r\right)}$ is a node-specific feature weighting vector and ⊙ denotes element-wise multiplication. The weighting vector emphasizes dimensions that are more informative for the current individual while suppressing unstable or low-value components. To avoid abrupt changes induced by short-term noise, the recalibration weights are updated using temporal smoothing:


$$\begin{array}{l} {\text{a}}_{i}^{\left(r\right)}=\beta {\text{a}}_{i}^{\left(r-1\right)}+\left(1-\beta \right){\hat{\text{a}}}_{i}^{\left(r\right)}, \end{array}$$ (30)

where ${\hat{\text{a}}}_{i}^{\left(r\right)}$ is estimated from recent local samples and β∈[0, 1) is a smoothing factor. This mechanism reinforces individual patterns while maintaining low computational overhead on the device side.

#### 4.3.3 Quality-aware aggregation and differential patch updating

If each edge node performs local adaptation independently, the system gradually loses the benefit of shared knowledge across devices. To preserve global consistency, the center aggregates local updates in a quality-aware manner. Let


$$\begin{array}{l} \text{Δ}\theta_{i}^{\left(r\right)}=\theta_{i}^{\left(r\right)}-\theta_{\text{meta}}^{\left(r\right)} \end{array}$$ (31)

denote the parameter increment uploaded by node *i* at round *r*. The center assigns an aggregation weight $w_{i}^{\left(r\right)}$ to each node according to data quality, task importance, state stability, and update credibility, and updates the shared prior as


$$\begin{array}{l} \theta_{\text{meta}}^{\left(r+1\right)}=\theta_{\text{meta}}^{\left(r\right)}+\overset{N}{\underset{i=1}{\sum }}w_{i}^{\left(r\right)}\text{Δ}\theta_{i}^{\left(r\right)},\;\overset{N}{\underset{i=1}{\sum }}w_{i}^{\left(r\right)}=1. \end{array}$$ (32)

Compared with simple averaging, this weighted aggregation is more robust because it suppresses contamination from noisy or low-confidence local updates.

After aggregation, the center does not return the full updated model. Instead, it constructs a compressed differential patch that contains only the most relevant parameter corrections for the target node:


$$\begin{array}{l} P_{i}^{\left(r\right)}=C\left(\theta_{\text{meta}}^{\left(r+1\right)}-\theta_{i}^{\left(r\right)}\right), \end{array}$$ (33)

where C(·) is a compression mapping that preserves only the critical updated components, such as important leaves, channels, or low-rank increments. The local node then fuses the patch with its current parameters using a controlled step size:


$$\begin{array}{l} \theta_{i}^{\left(r+1\right)}=\theta_{i}^{\left(r\right)}+\alpha_{i}^{\left(r\right)}P_{i}^{\left(r\right)}, \end{array}$$ (34)

where $\alpha_{i}^{\left(r\right)}$ controls the strength of patch fusion. Larger values are used when the local model diverges from the shared prior; smaller values protect a stable local model from unnecessary perturbation. Patch return reduces bandwidth because only selected corrections are transmitted.

#### 4.3.4 Event-driven synchronization

State and resource conditions determine when communication is triggered. Let $d_{i}^{\left(r\right)}$ denote the drift score of node *i* at round *r*, $g_{i}^{\left(r\right)}$ denote a risk-state gate, and $c_{i}^{\left(r\right)}$ denote whether local resources allow updating. Synchronization occurs only when drift or risk status justifies communication and the current device state permits execution. The trigger is defined as


$$\begin{array}{l} s_{i}^{\left(r\right)}=𝕀\left(d_{i}^{\left(r\right)}>\tau_{d}\vee g_{i}^{\left(r\right)}=1\right)\cdot 𝕀\left(c_{i}^{\left(r\right)}=1\right), \end{array}$$ (35)

where *𝕀*(·) denotes the indicator function, τ_*d*_ is the drift threshold, and $s_{i}^{\left(r\right)}=1$ indicates that synchronization is activated for node *i* at round *r*.

The event-driven policy avoids routine upload when the local model remains stable. Drift or high-risk status can still activate center-side correction, allowing the system to respond to clinically relevant change without repeated full synchronization.

Meta-initialization, regularized local updating, feature recalibration, weighted aggregation, differential patch return, and event-driven synchronization form the personalization loop. The deployed predictor can incorporate new individual evidence while keeping edge-side computation and communication bounded.

### 4.4 Edge collaborative optimization architecture

Feature distillation defines how stress-related information is represented, and incremental learning defines how the predictor changes over time. Stable clinical deployment also requires coordination of sensing, communication, aggregation, and intervention under latency, bandwidth, privacy, and workload constraints. The edge collaborative optimization architecture provides this coordination through compact summary transmission, adaptive aggregation, closed-loop dispatch, and resource-aware task allocation. The compact-summary interface, transmission constraints, aggregation, dispatch, task allocation, and low-load update rule are specified in Equations 36–45.

#### 4.4.1 Privacy-constrained edge summary transmission

The proposed system replaces continuous raw-stream upload with edge-center interaction in a compact edge summary space $F_{e}$. In this study, this privacy-constrained design is evaluated through the size of transmitted feature summaries and the avoidance of raw-stream upload; cryptographic protocols, reconstruction attacks, and formal differential-privacy guarantees are outside the present empirical scope. Let **z**_*i, t*_ denote the distilled edge-side feature representation produced by node *i* at time *t*. The transmitted summary is defined as


$$\begin{array}{l} {\text{f}}_{i,t}=\phi_{e}\left({\text{z}}_{i,t}\right),\;{\text{f}}_{i,t}\in F_{e}, \end{array}$$ (36)

where ϕ_*e*_(·) denotes the summary mapping and $F_{e}$ denotes the compressed communication space. This interface supplies inputs for aggregation, drift analysis, and dispatch without exposing raw physiological trajectories.

For prospective deployment, transmission is conditioned on anonymization and secure-channel requirements. Let *A*_*i*_ denote the transmission admissibility indicator for node *i*, and let *C*_*i, t*_ denote the communication-channel state. The admissibility condition is defined as


$$\begin{array}{l} A_{i}=1\;\Leftrightarrow \;\left({\text{f}}_{i,t}\;\text{is anonymized}\right)\wedge \left(C_{i,t}\;\text{is secure}\right), \end{array}$$ (37)

so a prospective deployment permits summary upload only when reconstruction risk and channel security have been addressed by the deployment environment. In the present retrospective evaluation, these terms serve as design requirements for the communication interface. The implemented evidence is the reduction of raw-stream transmission and the use of compact feature summaries; formal reconstruction-risk testing and secure-channel validation are reserved for deployment-stage security evaluation.

To characterize communication cost, the uplink load of node *i* over time interval T is defined as


$$\begin{array}{l} B_{i}\left(T\right)=\underset{t\in T}{\sum }|{\text{f}}_{i,t}|, \end{array}$$ (38)

where **f**_*i, t*_ denotes the byte size of the transmitted summary. Because **f**_*i, t*_ is generated from distilled low-dimensional features, the resulting uplink burden is substantially lower than that of continuous raw-data transmission.

#### 4.4.2 Adaptive model aggregation and closed-loop dispatch

After receiving summary information and local parameter increments from multiple edge nodes, the center must balance shared global knowledge against persistent individual differences. To this end, the architecture introduces a node-weighted aggregation matrix **W**_*v*_. Let $\text{Δ}\theta_{i}^{\left(r\right)}$ denote the parameter increment uploaded by node *i* at update round *r*, and let $w_{i}^{\left(r\right)}$ denote its aggregation weight. The weight matrix is defined as


$$\begin{array}{l} {\text{W}}_{v}^{\left(r\right)}=\text{diag}\left(w_{1}^{\left(r\right)},w_{2}^{\left(r\right)},\ldots ,w_{N}^{\left(r\right)}\right), \end{array}$$ (39)

and the global aggregation step is written as


$$\begin{array}{l} \theta_{\text{meta}}^{\left(r+1\right)}=\theta_{\text{meta}}^{\left(r\right)}+\overset{N}{\underset{i=1}{\sum }}w_{i}^{\left(r\right)}\text{Δ}\theta_{i}^{\left(r\right)},\;\overset{N}{\underset{i=1}{\sum }}w_{i}^{\left(r\right)}=1. \end{array}$$ (40)

The aggregation weights depend on node data quality, prediction confidence, and recent state stability, enabling robust weighted integration.

The center further embeds prediction output into a closed-loop dispatch process. Let *s*_*i, t*_ denote the risk score at the window or event level, and let *c*_*i, t*_ denote the current clinical context, including shift status, workload intensity, and recent alert frequency. The intervention action is generated by a clinical decision tree $T_{c}$:


$$\begin{array}{l} a_{i,t}=T_{c}\left(s_{i,t},c_{i,t}\right). \end{array}$$ (41)

A risk score is interpreted with operational context before staff adjustment, monitoring escalation, or other intervention recommendations are generated. The dispatch process remains executable, auditable, and workflow-oriented.

To avoid excessive synchronization during high-load clinical periods, the aggregation interval is adjusted according to the current workload:


$$\begin{array}{l} \tau_{r}=\tau_{\min}+\kappa l_{r}, \end{array}$$ (42)

where τ_min_ is the minimum aggregation interval, *l*_*r*_ is the clinical load indicator at round *r*, and κ is a scaling coefficient. Higher workload leads to longer aggregation intervals, whereas lower workload allows more timely assimilation of edge-side updates. This design keeps the collaborative loop active without turning synchronization into a source of operational instability.

#### 4.4.3 Resource-coordinated optimization

Real-time performance depends on both classifier cost and task placement across the edge node, the center, and nearby edge servers. The architecture uses hierarchical task allocation: feature extraction, window-level screening, and local alert triggering remain on the device; long-term trend analysis and cross-node model refinement are assigned to the center; intermediate-complexity tasks can be offloaded to nearby edge servers when the local node is overloaded or in a high-pressure state.

Let $Q=\left\{q_{1},\ldots ,q_{M}\right\}$ denote the task set, and let each task *q*_*m*_ be associated with computation cost, latency penalty, and priority. The task assignment function is defined as


$$\begin{array}{l} \pi \left(q_{m}\right)\in \left\{\text{edge},\text{center},\text{near-edge}\right\}. \end{array}$$ (43)

Resource allocation is then determined by


$$\begin{array}{l} \underset{\pi }{\min}\overset{M}{\underset{m=1}{\sum }}\left(\alpha_{c}c_{m}\left(\pi \left(q_{m}\right)\right)+\alpha_{t}d_{m}\left(\pi \left(q_{m}\right)\right)-\alpha_{p}p_{m}\right), \end{array}$$ (44)

where *c*_*m*_(·) denotes computation cost, *d*_*m*_(·) denotes latency penalty, *p*_*m*_ denotes task priority, and α_*c*_, α_*t*_, α_*p*_ are trade-off coefficients. Latency-sensitive high-priority tasks are assigned to the edge, and high-cost tasks with weaker real-time constraints are shifted to the center or near-edge servers.

This allocation rule supports an event-responsive operating mode. The system does not activate the full high-cost processing chain for all incoming data. Lightweight screening remains the default path, and heavier computation is triggered only around stress transitions or high-risk states. To prevent background updating from interfering with foreground monitoring, model update and patch fusion are further restricted to low-load windows. Let *L*(*t*) denote the clinical task load at time *t*. The update permission indicator is defined as


$$\begin{array}{l} m\left(t\right)=𝕀\left(L\left(t\right)<\tau_{L}\right), \end{array}$$ (45)

where τ_*L*_ is the low-load threshold. Batch update, patch fusion, and background maintenance are executed only when *m*(*t*) = 1. In practice, this condition corresponds to relatively low-intensity workflow segments, such as handoff intervals or other temporary load-reduction periods.

The edge collaborative optimization architecture links summary transmission, robust aggregation, intervention mapping, and dynamic task allocation in one operating logic. The edge provides fast response, the center provides stable model evolution, and nearby edge servers provide elastic buffering for intermediate workloads.

### 4.5 Study design and evaluation protocol

#### 4.5.1 Implementation boundary

The multi-node patch exchange, robust aggregation, task dispatch, and privacy-aware summary interfaces described above define the intended edge–center implementation boundary. The empirical evaluation instantiates this boundary through a calibrated 15D/48D cascade, held-out stream evaluation, edge-equivalent runtime tests, and a chronological feedback audit for personalized updating. Live multi-node hospital deployment, real-time patch exchange, and clinical dispatch are deployment-stage extensions of this boundary.

#### 4.5.2 Deployment environment

Experiments were conducted in a hybrid edge–center setting consistent with the method design. The edge side represents wearable devices used for continuous nurse monitoring and performs data reception, lightweight feature extraction, front-stage screening, and local alert triggering. The center side is represented empirically by model initialization, threshold selection, and the chronological personalization audit. Runtime tests used a single-core, single-thread, 1.2 GHz edge-equivalent software setting, with physical smartwatch or patch deployment reserved for implementation-stage validation. Deployment targets were therefore defined by end-to-end latency, CPU duty cycle, memory footprint, and uplink traffic. Prediction latency was required to remain below 100 ms and CPU usage below 15%. Direct battery drain and hardware power consumption were not measured.

#### 4.5.3 Datasets and task definition

The evaluation used WESAD as the public benchmark and a real-world continuous nurse-monitoring dataset for stream-level alert analysis (57–60). WESAD contains 15 subjects in the processed protocol and was used for subject-wise window-level risk prediction, with 12 subjects for training and 3 unseen subjects for testing. Each window received a risk score and was evaluated as a binary high-risk or non-high-risk decision after threshold selection.

The nurse-monitoring dataset contains 15 nurses and 596 wearable recording sessions in the processed feature files. The available wearable channels used in the present feature pipeline were electrodermal activity (EDA), heart rate/heart-rate variability derived from HR/IBI, and skin temperature; other physiological variables listed in the system model, such as blood pressure, oxygen saturation, respiration, and tri-axial acceleration, describe the general NEST sensing interface but were not available in both evaluated datasets and were not used in the final nurse-monitoring feature pipeline. The accompanying survey file contained 358 self-reported stress intervals with start time, end time, date, stress level, and stressor categories, including COVID-related care, treating a COVID patient, patient crisis, patient or family interaction, doctors or colleagues, ancillary services, increased workload, technology-related stress, lack of supplies, documentation, competency-related stress, safety threats, and physical or workflow environment. Department, shift-type, detailed inclusion/exclusion criteria, and ward-level monitoring-duration variables were not available in the public metadata used for this secondary analysis, and therefore were not inferred or used as covariates. Stress levels were parsed as low, medium, or high when available; medium- and high-stress intervals were treated as positive stress events for event-level evaluation.

The nurse-monitoring data were split by subject using a fixed seed, with 12 nurses used for model fitting and calibration and 3 held-out nurses used only for final stream-level testing. Raw survey events numbered 358 before filtering. For event-level testing, events were restricted to the held-out test nurses and to medium/high stress levels, yielding 24 positive test events. Window labels were assigned by temporal overlap with labeled stress intervals, and thresholded window outputs were aggregated into alert events through the cascade and temporal-control rules. This setup keeps the window-level and event-level tasks within the same deployment pipeline while preventing test-subject information from being used during model fitting or threshold selection.

The leakage-control procedure was therefore implemented at three points. First, subject identity defined the main train/test boundary, so windows from the three held-out nurses were not mixed into model fitting. Second, calibration, imputation medians, feature selection, and threshold selection were derived from the training partition or from training-subject out-of-fold predictions. Third, final event-level metrics were computed only after applying the locked model and locked thresholds to the held-out continuous streams. The supplementary 199-event analysis follows the same principle by evaluating training subjects only through out-of-fold predictions and keeping held-out subjects as independent test predictions.

#### 4.5.4 Feature representation and model configuration

Two feature sets instantiate the compact representation design: a 48-dimensional set for confirmation and offline reference, and a 15-dimensional set for lightweight edge screening. Table 1 reports the retained feature groups and the exact 15D screening variables so that the implemented representation is reproducible.

**Table 1:** Implemented 48D confirmation and 15D screening feature representations.

Representation	Feature groups	Retained variables
48D confirmation/offline reference	EDA, temperature, HR/IBI, and lagged means	EDA mean, minimum, maximum, standard deviation, skewness, kurtosis, peak count; temperature mean, minimum, maximum, standard deviation, skewness, kurtosis; HR mean, minimum, maximum, standard deviation, skewness, kurtosis, RMSSD, IBI count; lagged EDA, temperature, and HR means from lags 1–9
15D lightweight screening	Compact EDA, temperature, HR/IBI, and selected lagged means	EDA mean, minimum, maximum, peak count; temperature mean; HR mean; RMSSD; lag-1 and lag-2 EDA means; lag-1 and lag-2 HR means; lag-1 and lag-2 temperature means; lag-5 EDA mean; lag-5 HR mean

Missing feature values were imputed using medians estimated from the training partition, and the retained 15D/48D feature lists were fixed before held-out test evaluation. Random forests served as base classifiers. Window-level outputs were processed by cross-fit estimation, sigmoid calibration, and threshold selection. In the clinical nurse-monitoring experiments, the fixed subject-wise split used 12 nurses for fitting, calibration, and threshold selection, and the 3 held-out nurses were used only for final stream-level event testing. Threshold tuning was performed on training-derived validation subjects or out-of-fold predictions; the held-out test subjects were not used to choose thresholds. Event-level evaluation used a two-stage cascade in which the 15D model screened frequent windows and the 48D model confirmed triggered candidates. Bilinear interaction and dynamic-importance equations in the method section motivate the compact-representation design; the reported experiments instantiate this design through fixed 15D/48D feature sets and a calibrated cascade.

#### 4.5.5 Evaluation metrics

The experiments were evaluated using window-level metrics, including Accuracy, F1, ROC-AUC, PR-AUC, Precision, Recall, and Specificity. In addition, event-level and deployment-level metrics were used to assess continuous monitoring behavior and implementation efficiency, including Event Recall, False Alerts/h, Alerts/h, latency, CPU duty cycle, memory usage, and upload traffic. The primary event-level test follows a strict subject-wise held-out stream protocol and contains 24 positive events after the predefined subject and stress-level filtering. Event recall is therefore reported together with integer hit counts and 95% Wilson binomial confidence intervals, and supplementary out-of-sample and multi-split analyses are used to assess stability without changing the primary held-out test definition.

## 5 Results

### 5.1 Overview

The results are organized around four deployment questions: how much discrimination remains after 15D compression, how cascade alerting changes event-level alert burden, whether runtime and communication stay within edge budgets, and whether incremental updating improves behavior under individual drift.

### 5.2 Lightweight feature distillation and window-level classification results

**Figure 4:**
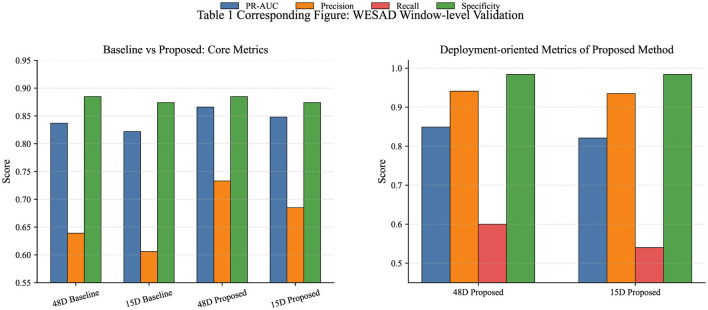
WESAD 48D/15D baseline vs proposed window-level comparison.

**Figure 5:**
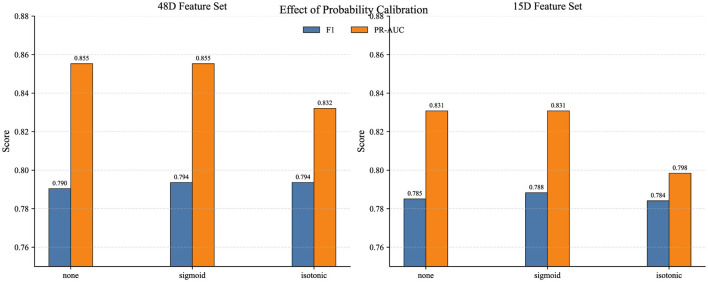
Comparison of different probability calibration schemes.

The baseline-versus-proposed comparison is visualized in Figure 4, and the effects of probability calibration are shown in Figure 5. This section reports the window-level results on WESAD. We examine whether the 15D distilled representation preserves the discriminative performance of the 48D feature space and whether probability calibration provides a stable basis for subsequent event-level alerting.

#### 5.2.1 Effectiveness of lightweight feature distillation

Table 2 reports the WESAD results for the 48D and 15D feature sets. With baseline random forest, the 48D representation achieved 0.837 Accuracy and 0.639 F1, while the 15D representation achieved 0.822 Accuracy and 0.606 F1. Cross-fit estimation, sigmoid calibration, and threshold selection raised the 48D result to 0.866 Accuracy and 0.733 F1, and the 15D result to 0.848 Accuracy and 0.685 F1. ROC-AUC values were 0.885 and 0.874, respectively, and both calibrated settings reached 0.984 Specificity.

**Table 2:** Window-level classification performance of the 48D and 15D feature sets on WESAD.

Feature set	Setting	Acc.	F1	ROC-AUC	Prec.	Spec.
48D	Baseline RF	0.837	0.639	0.885	–	–
15D	Baseline RF	0.822	0.606	0.874	–	–
48D	Cross-fit + sigmoid calib + τ^*^	0.866	0.733	0.885	0.941	0.984
15D	Cross-fit + sigmoid calib + τ^*^	0.848	0.685	0.874	0.935	0.984

τ^*^ denotes the validation-selected optimal decision threshold.

The 48D-to-15D reduction caused limited degradation relative to the compression ratio: Accuracy decreased by 0.018, F1 by 0.048, and ROC-AUC by 0.011. The calibrated 15D model still reached 0.935 Precision, close to the 0.941 value of 48D, with the same 0.984 Specificity. These values justify using 15D as the screening branch, where lower feature-processing and transmission cost outweigh the small loss relative to the full feature set.

#### 5.2.2 Probability calibration and threshold stability

It should be noted that Table 2 reports the main subject-wise held-out evaluation, whereas Tables 3, 4 report the calibration and operating-point analysis under the threshold-selection validation setting. These values are therefore used for different purposes and should not be interpreted as repeated estimates on the same test split. The usefulness of the window-level classifier in continuous monitoring depends not only on ranking quality but also on the stability of the selected operating point. Table 3 compares three calibration schemes, namely none, sigmoid, and isotonic, for both the 48D and 15D feature sets. Sigmoid calibration yields small but consistent improvements on both representations. For the 48D feature set, Accuracy increases from 0.8807 to 0.8830, F1 from 0.7904 to 0.7936, Precision from 0.8530 to 0.8605, and Specificity from 0.9442 to 0.9475. For the 15D feature set, Accuracy increases from 0.8769 to 0.8792, F1 from 0.7851 to 0.7883, Precision from 0.8409 to 0.8481, and Specificity from 0.9387 to 0.9420, while Recall remains unchanged for both feature sets.

**Table 3:** Window-level classification results under different probability calibration schemes.

Feature set	Calibration	τ^*^	Acc.	F1	Prec.	Spec.	ROC-AUC	PR-AUC
48D	None	0.25	0.8807	0.7904	0.8530	0.9442	0.8825	0.8553
48D	Sigmoid	0.23	0.8830	0.7936	0.8605	0.9475	0.8825	0.8553
48D	Isotonic	0.16	0.8830	0.7936	0.8605	0.9475	0.8723	0.8321
15D	None	0.26	0.8769	0.7851	0.8409	0.9387	0.8726	0.8308
15D	Sigmoid	0.21	0.8792	0.7883	0.8481	0.9420	0.8726	0.8308
15D	Isotonic	0.05	0.8761	0.7841	0.8385	0.9376	0.8544	0.7984

τ^*^ denotes the validation-selected optimal decision threshold.

**Table 4:** Window-level comparison of different classifiers on the 15D feature set (WESAD, sigmoid calibration).

Method	τ^*^	Accuracy	F1	Precision	Recall	Specificity	ROC-AUC	PR-AUC
RF	0.21	0.8792	0.7883	0.8481	0.7363	0.9420	0.8726	0.8308
LR	0.26	0.7728	0.6598	0.6080	0.7214	0.7954	0.8307	0.6169
RFDEEP	0.16	0.7044	0.6460	0.5093	0.8831	0.6258	0.8882	0.8383
GBDT	0.18	0.7751	0.6973	0.5920	0.8483	0.7429	0.9000	0.8167
NB	0.19	0.6793	0.5622	0.4822	0.6741	0.6816	0.7125	0.6335
XGB	0.20	0.7698	0.6659	0.5980	0.7512	0.7779	0.8969	0.8550
SVM	0.19	0.6033	0.6003	0.4336	0.9751	0.4398	0.9267	0.8577
MLP	0.17	0.5023	0.5511	0.3803	1.0000	0.2834	0.9426	0.8206
AdaBoost	0.18	0.8754	0.8136	0.7490	0.8905	0.8687	0.9400	0.8511

τ^*^ denotes the validation-selected optimal decision threshold.

The main advantage of sigmoid calibration lies in operating-point stability. The optimal thresholds remain in a moderate range, with τ^*^ = 0.23 for 48D and τ^*^ = 0.21 for 15D. By contrast, isotonic calibration does not improve the thresholded metrics on 48D and becomes noticeably less stable on the 15D representation, where the optimal threshold drops to 0.05 and the ranking metrics also deteriorate, with ROC-AUC and PR-AUC decreasing to 0.8544 and 0.7984, respectively. Given the need for a unified and stable thresholding scheme in the subsequent event-level analyses, sigmoid calibration was used in the main experiments.

#### 5.2.3 Window-level results under different classifiers

**Figure 6:**
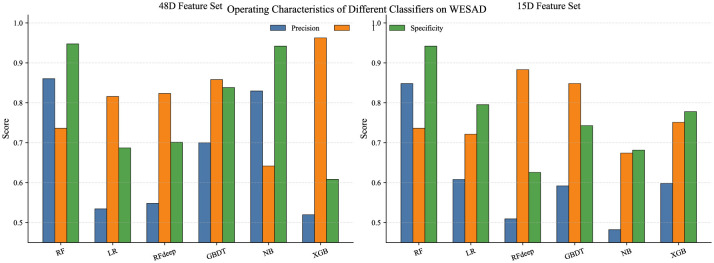
Operating-characteristic comparison of multiple classifiers under the 48D and 15D feature settings.

Additional comparisons were conducted under both 48D and 15D feature settings to examine classifier dependence. The benchmark set was expanded to cover linear, kernel, tree-ensemble, probabilistic, boosting, and neural-network families, including random forest (61), logistic regression (62), support-vector machine (63), gradient boosting (64), Gaussian naive Bayes (65), XGBoost (66), multilayer perceptron (67), and AdaBoost (68). Figure 6 presents the operating-characteristic comparison across the two feature settings, whereas Table 4 reports the detailed thresholded results for the 15D sigmoid-calibrated setting used in the lightweight screening branch.

The other classifiers show different failure modes in the 15D sigmoid-calibrated setting. RFDEEP, SVM, and MLP reached high Recall, but their Specificity dropped markedly, making them unsuitable for false-trigger control in a continuous stream. AdaBoost achieved the highest 15D F1 in this supplementary WESAD comparison, but the event-level nurse-monitoring analysis retained sigmoid-calibrated random forest because the selected cascade, alert-budget analysis, runtime tests, and personalization audit were all built around the fixed RF operating point before held-out stream evaluation. GBDT and XGBoost achieved strong ROC-AUC values, yet their thresholded Precision and Specificity were weaker than those of random forest. Because the cascade alert system depends on the selected operating point as well as ranking quality, sigmoid-calibrated random forest was retained for later analyses. The comparison across 48D and 15D settings further indicates that the observed behavior is associated with the distilled representation, calibration, and alert-control design.

The event-level analysis therefore uses the 15D model for screening, the 48D model for confirmation, and sigmoid-calibrated random forest as the common classifier setting. This ordering separates four factors that could otherwise be conflated: feature dimensionality is examined in the 48D/15D comparison, calibration is examined before event aggregation, classifier dependence is examined under the same 15D representation, and cascade behavior is examined after fixing the classifier and feature roles. The clinical window-level classifier table is used as a supplementary difficulty analysis for model selection under continuous monitoring. The event-level cascade results characterize two-stage alert control under a fixed calibrated classifier. Table 5 summarizes the relationship between previously published wearable-stress methods and the same-protocol baselines evaluated in this study.

**Table 5:** Relation to previously published wearable-stress methods and the same-protocol baselines used in this study.

Reference or baseline family	Dataset/setting	Model family	Comparison role in this study
Schmidt et al. (58); UCI WESAD (57)	WESAD wearable stress benchmark	Classical multimodal stress-recognition baselines	Source public benchmark; our WESAD split uses unseen subjects and reports window-level performance under a fixed protocol
Mishra et al. (26)	Physiological stress-detection reproducibility, including WESAD-style protocols	Reproducibility analysis of stress-detection pipelines	Supports same-protocol reruns when reported scores are not directly comparable
SELF-CARE (13)	Wearable stress detection with low-power edge constraints	Selective fusion and context-aware edge inference	Published edge-oriented stress detector used as methodological reference for compact edge inference
TinyStressNAS (53)	On-device stress detection	Automated feature/model search for constrained devices	Published on-device stress-recognition reference for lightweight deployment
Korkmaz et al. (28)	Nurse stress time-series sensor data	Comparative classifier evaluation	Nurse-stress algorithm reference; task definition differs from our continuous-stream alert protocol
Same-protocol reruns in this study	WESAD subject-wise split and held-out nurse streams	RF, LR, RFDEEP, GBDT, NB, XGBoost, SVM, MLP, AdaBoost	Direct numerical baselines under the same feature table, calibration logic, threshold selection, and event-alert protocol

### 5.3 Event-level alerting performance in real nurse monitoring scenarios

Event-level alerting was evaluated on real continuous nurse-monitoring data. The analysis focuses on the recall–alert burden trade-off under cascade operation and on the effects of alternative baselines, gate budget, and confirmation threshold.

#### 5.3.1 Main event-level results in the real scenario

Table 6 reports the main event-level comparison using the predefined primary held-out stream protocol and the selected cascade operating point. Single48 detected 14 of 24 held-out test events, giving an Event Recall of 0.58 with a 95% Wilson confidence interval (CI) of 0.39–0.76. Its false-alert and total-alert rates were 9.68 and 10.65 alerts per hour. This operating point keeps moderate sensitivity but generates an alert stream too dense for continuous nursing workflows.

**Table 6:** Main event-level comparison on real nurse monitoring data using the selected cascade operating point.

Method	Event recall (95% CI)	Hits/24	False alerts/h	Alerts/h
Single48 (sigmoid-RF)	0.58 [0.39, 0.76]	14 / 24	9.68	10.65
Cascade (gate_budget = 0.10, τ_2_ = 0.07)	0.58 [0.39, 0.76]	14 / 24	1.88	2.10
LR-48D	0.83 [0.64, 0.93]	20 / 24	26.42	27.96
RFDEEP-48D	0.79 [0.60, 0.91]	19 / 24	19.31	20.82
GBDT-48D	0.71 [0.51, 0.85]	17 / 24	13.17	14.24
XGBoost-48D	0.79 [0.60, 0.91]	19 / 24	16.99	18.36

The selected cascade operating point substantially changes this behavior without reducing the event hit count. Under the configuration (gate_budget = 0.10, τ_2_ = 0.07) with the selected temporal aggregation rule, the system also detected 14 of 24 events, corresponding to Event Recall of 0.58 (95% CI: 0.39–0.76), while reducing False Alerts/h to 1.88 and Alerts/h to 2.10. The event hit rate is maintained, and the false-alert burden and overall alert density are both reduced to approximately one fifth of the Single48 level. This cascade point therefore provides an alert-control operating point with the same observed hit count and a substantially lighter alert stream. Operationally, the same number of detected stress events in this split would require far fewer non-event alerts for nurses, managers, or occupational-health staff to review during continuous monitoring. The added value is a reduction in avoidable interruptions and alert fatigue while preserving the observed screening coverage in the primary held-out stream. Because the 24 positive events come from the strict independent held-out stream instead of event-level random sampling, the result prioritizes subject-level independence.

Integer hit counts, confidence intervals, bootstrap stability analysis, the 199-event out-of-sample analysis, and additional subject-level splits are reported in the following subsections to make the evidence transparent.

#### 5.3.2 Module-level ablation of the cascade

**Figure 7:**
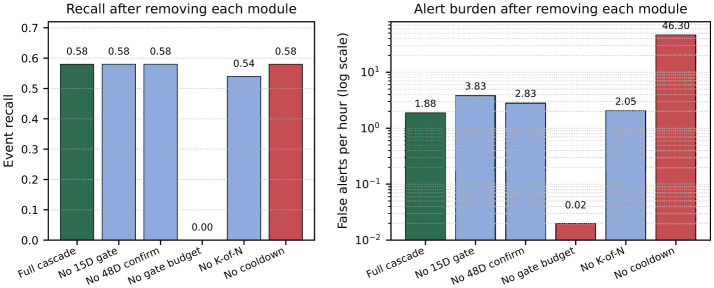
Module-level ablation summary for the selected cascade. The left panel compares event recall after removing each module, and the right panel compares false-alert density on a logarithmic scale.

A module-level ablation was conducted on the same held-out nurse-monitoring streams to separate cascade behavior from classifier choice and threshold selection. The full operating point used the 15D screening gate, the 48D confirmation model, adaptive gate budgeting (gate_budget = 0.10), fixed confirmation threshold τ_2_ = 0.07, a 2-of-3 temporal confirmation rule, and a 300 s cooldown. Each ablation removed one component while keeping the remaining settings fixed, except for the temporal-control rows where the corresponding rule was explicitly disabled. The module-level effects on event recall and false-alert density are visualized in Figure 7.

Table 7 shows that the 15D gate, 48D confirmation, adaptive gate budget, and cooldown play distinct roles. Removing the 15D gate preserved the same 14/24 event hit count but forced every window through the 48D branch and doubled false-alert density from 1.88 to 3.83 per hour. Removing 48D confirmation also preserved 14/24 hits but increased false alerts to 2.83 per hour, indicating that the confirmation stage filters many gate-triggered false positives. Removing adaptive gate budgeting made the fixed gate overly conservative in this held-out stream, reducing the gate ratio to 0.001 and collapsing event recall to 0/24. Removing the K-of-N rule had a smaller effect, lowering hits from 14 to 13 and increasing false alerts by 9.0%; this rule is retained as a smoothing safeguard. Removing cooldown caused alert density to become clinically unusable, with false alerts rising to 46.30 per hour. These results indicate that the gate controls expensive-stage usage, the confirmation model reduces false positives, the budget prevents gate collapse, and cooldown prevents repeated alerts from the same high-risk episode.

**Table 7:** Module-level ablation of the selected event-trigger cascade on the held-out nurse-monitoring streams.

Variant	Deleted component	Hits/24	Event recall (95% CI)	False alerts/h	Alerts/h	48D Invocation ratio	Main effect
Full cascade	–	14 / 24	0.58 [0.39, 0.76]	1.88	2.10	0.102	Reference operating point
No 15D gate	15D screening gate	14/24	0.58 [0.39, 0.76]	3.83	4.19	1.000	Same hits, but false alerts doubled and all windows invoke 48D
No 48D confirmation	48D confirmation	14/24	0.58 [0.39, 0.76]	2.83	3.08	0.102	Gate-only stream produces more false positives
No gate budget	Adaptive gate budget	0/24	0.00 [0.00, 0.14]	0.02	0.02	0.001	Fixed gate is too conservative and recall collapses
No K-of-N	Temporal confirmation	13/24	0.54 [0.35, 0.72]	2.05	2.27	0.102	Modest smoothing benefit; retained as safeguard
No cooldown	Cooldown suppression	14/24	0.58 [0.39, 0.76]	46.30	49.74	0.102	Repeated alerts become clinically unusable

#### 5.3.3 Stability and supplementary event sensitivity analyses

##### 5.3.3.1 Bootstrap stability

To further assess the stability of the event-level finding under the small test-event count, we performed a non-parametric event-level bootstrap analysis with 10,000 resamples of the 24 held-out events and also computed exact binomial confidence intervals. Table 8 shows wide intervals for both Single48 and Cascade, confirming that event recall is statistically imprecise at this sample size even when the observed hit counts are identical. The delay analysis further shows the cost of cascade confirmation. Median first-hit delay increases from 11.5 s for Single48 to 298.5 s for Cascade, while P95 delay increases from 1078.4 s to 1528.6 s. Thus, the selected cascade improves alert sparsity while preserving the observed event hit count in this split, but it also delays first confirmation for detected events.

**Table 8:** Event-level bootstrap stability analysis on the 24 held-out positive stress events.

Method	Hits/24	Recall	Exact 95% CI	Bootstrap 95% CI	Median delay (s)
Single48	14/24	0.58	[0.37, 0.78]	[0.38, 0.79]	11.5
Cascade	14/24	0.58	[0.37, 0.78]	[0.38, 0.79]	298.5

##### 5.3.3.2 All-subject out-of-sample event check

Because the strict fully held-out primary split contains 24 positive events, an all-subject out-of-sample analysis was conducted without redefining the main test. Table 9 evaluates the 12 training subjects only through subject-level out-of-fold predictions from the cross-fitting procedure, while the three held-out subjects retain their independent test predictions. This design increases the event count from 24 to 199 without evaluating any subject on predictions from a model trained on that same subject. Single15 and Single48 reach similar recall on the expanded event set, but both produce dense alert streams. The selected cascade reduces False Alerts/h from approximately 16.2 to 3.56 and Alerts/h from approximately 17.8 to 4.07, at the cost of lower recall in this supplementary analysis.

**Table 9:** Supplementary all-subject out-of-sample event analysis using training-subject out-of-fold predictions plus held-out test-subject predictions.

Method	Event recall (95% CI)	Hits/199	False alerts/h	Alerts/h	Gate ratio
Single15 (sigmoid-RF)	0.80 [0.74, 0.85]	159/199	16.27	17.97	–
Single48 (sigmoid-RF)	0.80 [0.74, 0.85]	159/199	16.19	17.83	–
Cascade (gate_budget = 0.10, τ_2_ = 0.07)	0.62 [0.55, 0.69]	124 / 199	3.56	4.07	0.104

##### 5.3.3.3 Additional subject-level splits

Table 10 evaluates RF training and event-level performance on two additional subject-level splits. These additional held-out splits increase the range of evaluated test events and show that cascade consistently reduces alert density, while the associated recall behavior varies by split.

**Table 10:** Supplementary multi-split held-out event sensitivity using RF models trained from the clinical feature table.

Split seed	Test subjects	Method	Hits/events	Recall (95% CI)	False alerts/h	Alerts/h
2025	7E, BG, CE	Single48	14/24	0.58 [0.39, 0.76]	9.68	10.65
2025	7E, BG, CE	Cascade	14/24	0.58 [0.39, 0.76]	1.88	2.10
2026	E4, F5, 83	Single48	60/70	0.86 [0.76, 0.92]	26.05	28.40
2026	E4, F5, 83	Cascade	45/70	0.64 [0.53, 0.74]	3.45	4.16
2027	6D, 15, BG	Single48	16/33	0.48 [0.33, 0.65]	15.24	16.00
2027	6D, 15, BG	Cascade	10/33	0.30 [0.17, 0.47]	4.24	4.42

#### 5.3.4 Comparison with alternative methods

The nurse-monitoring dataset has been introduced as a public dataset for continuous hospital stress monitoring (59, 60), and prior wearable-stress studies have reported window-level or sequence-level classifiers on WESAD and related physiological datasets (13, 26, 28, 58). Direct event-level comparison is limited because most prior methods do not report continuous-stream alert counts, cooldown behavior, or false alerts per hour on the same nurse-monitoring event protocol. The present analysis therefore compares alternative classifiers and non-cascade/cascade operating points re-evaluated on the same held-out nurse-monitoring streams, while using prior published methods as methodological references for wearable stress recognition and edge-oriented stress detection. The same-protocol event-level baselines support comparison of alert metrics within the present evaluation protocol.

**Figure 8:**
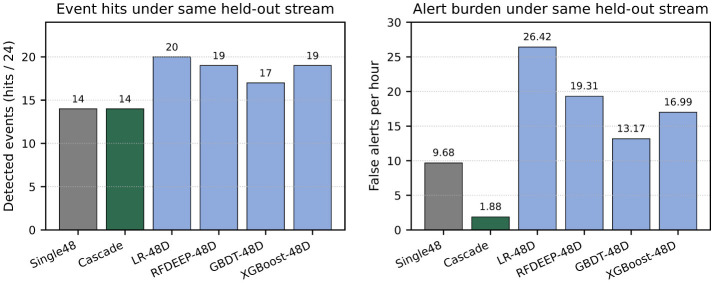
Event-level comparison of completed same-protocol classifier and cascade operating points on the primary held-out nurse-monitoring streams.

The comparison with alternative completed classifier runs confirms that event-level quality cannot be judged by recall alone. LR-48D attains 20 hits out of 24 events, RFDEEP-48D and XGBoost-48D each attain 19 hits out of 24 events, and GBDT-48D attains 17 hits out of 24 events. These higher hit counts are accompanied by markedly heavier alert burdens. False Alerts/h rise to 26.42 for LR-48D, 19.31 for RFDEEP-48D, 13.17 for GBDT-48D, and 16.99 for XGBoost-48D, while Alerts/h increase to 27.96, 20.82, 14.24, and 18.36, respectively. The selected RF cascade detects 14 of 24 events and has the lowest false-alert density among these completed operating points, lowering False Alerts/h to 1.88 and Alerts/h to 2.10. Figure 8 visualizes this trade-off: more aggressive 48D classifiers detect more held-out events, whereas the selected cascade provides the sparsest alert stream among the compared operating points. These results show that the cascade contribution is primarily alert-density control after a candidate high-risk stream has been formed.

Given the small number of held-out positive events, the confidence intervals are wide. More aggressive baselines detected more events, but they also produced alert rates that would be difficult to manage in continuous monitoring. The selected cascade point is therefore an alert-control operating point that preserved the Single48 hit count in the primary split while substantially reducing alert density. Its main contribution is the same observed hit count with lower alert density. Additional cascade settings are examined below to separate the effect of the gate budget from the confirmation threshold.

#### 5.3.5 Joint effect of gate budget and confirmation threshold

**Figure 9:**
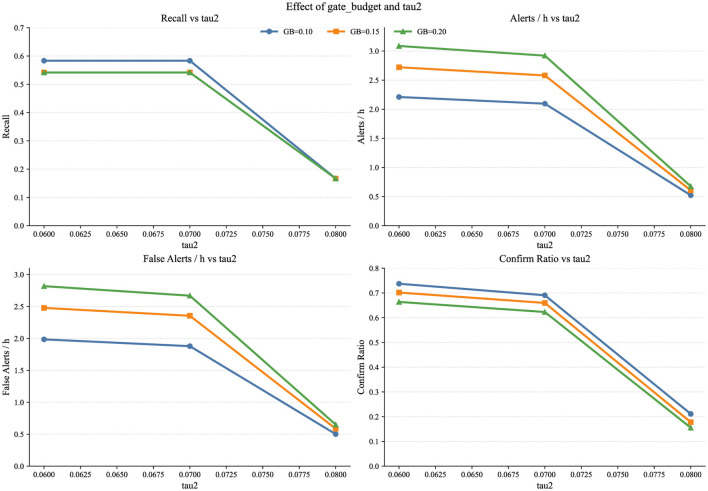
Four-panel comparison under different gate_budget and τ_2_ settings.

The joint effects of gate budget and confirmation threshold are visualized in Figure 9. Cascade behavior depends on both the front-stage gate budget and the back-stage confirmation threshold. Table 11 shows two distinct effects. Tightening τ_2_ from 0.06 to 0.07 reduces alert burden without changing recall at each tested budget level. At gate_budget = 0.10, Event Recall and Hits/24 remain 0.58 and 14, while False Alerts/h falls from 1.99 to 1.88 and Alerts/h from 2.21 to 2.10. Raising τ_2_ further to 0.08 makes the confirmation stage overly conservative, detecting only 4 of 24 events under all tested budgets. Thus, τ_2_ directly controls the sensitivity–sparsity balance.

**Table 11:** Event-level performance under different combinations of gate budget and confirmation threshold.

gate_budget	τ_2_	Event recall	Alerts/h	False alerts/h	Gate ratio	Confirm ratio
0.10	0.06	0.58	2.21	1.99	0.102	0.737
0.10	0.07	0.58	2.10	1.88	0.102	0.691
0.10	0.08	0.17	0.52	0.50	0.102	0.211
0.15	0.06	0.54	2.72	2.48	0.155	0.702
0.15	0.07	0.54	2.58	2.36	0.155	0.660
0.15	0.08	0.17	0.61	0.59	0.155	0.178
0.20	0.06	0.54	3.09	2.82	0.205	0.664
0.20	0.07	0.54	2.92	2.67	0.205	0.623
0.20	0.08	0.17	0.68	0.65	0.205	0.156

The budget parameter controls how many candidate windows reach confirmation. Increasing gate_budget from 0.10 to 0.20 raises the Gate Ratio from about 0.102 to 0.205 and increases alert density, but does not improve recall in this sweep. The selected point, gate_budget = 0.10 and τ_2_ = 0.07, preserves the Single48 hit count of 14/24 while giving the lowest alert density among the non-collapsed settings. This sweep therefore separates the cascade effect from the classifier effect and exposes a tunable operating surface for selecting an alert-density operating point.

The selected cascade configuration is therefore used in the deployment analysis because it preserves the Single48 hit count in the primary split while reducing false alerts and total alert density.

### 5.4 Edge-side real-time performance, resource overhead, and communication load

This section reports the deployment-level results of the proposed framework, including runtime latency, resource usage, and communication load under the edge-center collaborative setting.

#### 5.4.1 Edge-side real-time performance and resource usage

Table 12 reports the runtime statistics for three test subjects under the 15D gate path and the 48D confirmation path. The P95 end-to-end latency of the 15D gate path is 3.0447 ms, 3.0381 ms, and 3.2832 ms for subjects 7E, BG, and CE, respectively. For the 48D confirmation path, the corresponding values are 10.8742 ms, 4.9682 ms, and 11.8639 ms. All measured latencies remain in the millisecond range and stay well below the 100 ms deployment constraint for both the lightweight screening path and the higher-information confirmation path.

**Table 12:** Edge-side real-time latency and memory overhead on different test subjects.

Subject	Path	*t*_TOTAL_ (P95, ms)	RSS (P95, MB)
7E	15D gate	3.0447	120.4063
7E	48D confirm	10.8742	122.2461
BG	15D gate	3.0381	104.9297
BG	48D confirm	4.9682	103.9922
CE	15D gate	3.2832	121.0781
CE	48D confirm	11.8639	122.1563

Memory usage remains stable across subjects and paths. The P95 RSS values range from approximately 104 MB to 122 MB, with no evidence of substantial resource inflation caused by the two-stage structure. In the all-subject summary, the P95 CPU duty cycle is 6.77% for both the 15D gate path and the 48D confirmation path, while the average CPU occupancy is only 3.06% and 3.28%, respectively. These values remain well below the 15% system-level constraint, with no evidence of persistent compute saturation on the edge side during long-duration online monitoring.

#### 5.4.2 Single-node communication load

Uplink traffic is a direct constraint for wearable edge devices because it affects bandwidth demand, energy use, and center-side receiving load. Table 13 compares raw-stream upload, 48D feature upload, and 15D feature upload. Raw-stream transmission requires 921,600 bytes per hour. The 48D feature representation reduces the load to 138,240 bytes per hour, or 15.0% of the raw-stream level. The 15D representation reduces it further to 43,200 bytes per hour, or 4.69% of the raw-stream baseline.

**Table 13:** Communication load comparison among raw stream, 48D features, and 15D features.

Scheme	Bytes/stride	Bytes/min	Bytes/hour	Relative to raw
Raw stream	1,280	15,360	921,600	1.0000
Features-48D	192	2,304	138,240	0.1500
Features-15D	60	720	43,200	0.0469

This communication pattern follows directly from the cascade design. Most windows pass through the compact 15D path, and only candidate windows activate the 48D confirmation branch. The reduction is therefore not a standalone compression effect; it comes from pairing low-dimensional features with budget-controlled triggering.

#### 5.4.3 Communication and trigger load under multiple nodes

Scalability was evaluated by simulating communication load under increasing edge-node counts. Table 14 compares Raw Stream, Always-48D, and Cascade settings for 1, 5, 10, and 15 nodes. Total upload volume increases in all schemes, but not at the same rate. At 15 nodes, Raw Stream reaches 13824000 bytes per hour and Always-48D reaches 2,073,600 bytes per hour, whereas Cascade requires 873673.91 bytes per hour. The Cascade load corresponds to approximately 6.32% of Raw Stream and 42.13% of Always-48D.

**Table 14:** Communication and trigger-load simulation under different edge-node scales.

*n*_nodes_	Raw/h	Always-48D/h	Cascade/h	Trigger win./h	Bytes/4 h
1	921,600	138,240	58244.93	113.98	232979.71
5	4,608,000	691,200	291224.64	569.88	1164898.54
10	9,216,000	1,382,400	582449.27	1139.77	2329797.08
15	13,824,000	2,073,600	873673.91	1709.65	3494695.62

Trigger load grows in a controlled manner. Trigger Windows/h increases from 113.98 at one node to 1709.65 at fifteen nodes, and bytes per 4-h cycle increase from 232979.71 to 3494695.62. The near-linear increase indicates that the confirmation burden remains predictable as deployment scale expands. Predictable growth is important for ward-level or multi-shift monitoring, where communication planning and storage budgets must remain manageable as more devices are added.

The deployment results keep the selected 15D/48D cascade within the latency, CPU, memory, and communication budgets used in this study. The same configuration is used for temporal-control and personalization analysis. Table 15 reports window-level performance after chronological personalized updating, Table 16 presents the protocol ablation, and Table 17 verifies the subject-level separation of adaptation and evaluation windows.

**Table 15:** Window-level performance after leakage-controlled personalized incremental updating.

Setting	Method	Acc.	F1	Prec.	Recall	Spec.
Prefix feedback	Baseline before update	0.305	0.131	0.071	0.806	0.271
Prefix feedback	Updated + fixed threshold	0.144	0.131	0.070	0.995	0.085
No-update control	Baseline/incremental identical	0.305	0.131	0.071	0.806	0.271

**Table 16:** Ablation of the chronological personalization protocol on held-out clinical test subjects.

Setting	Subjects	Adapt ratio	ΔF1	ΔRecall	ΔSpec.
No update control (adapt_ratio = 0)	3	0.0	0.000	0.000	0.000
Prefix feedback, fixed post-update threshold	3	0.2	0.000	+0.189	–0.186

**Table 17:** Subject-level check of the chronological adaptation/evaluation split in the incremental-update experiment.

Subject	Adapt used	Pos. adapt	Eval windows	Update	Baseline F1	Updated F1
7E	864	216	7,241	Yes	0.098	0.111
BG	952	238	10,737	Yes	0.062	0.098
CE	9,364	2,341	15,893	Yes	0.176	0.158

### 5.5 Temporal behavior control and personalization

The preceding sections established the main performance profile of the proposed framework at the window level, the event level, and the deployment level. The remaining questions are whether the alerting process admits interpretable temporal control and whether the lightweight edge-side model can adapt to individual drift through incremental updating. This section addresses these two issues through supplementary experiments on temporal behavior and personalization.

#### 5.5.1 Supplementary operating-point analysis

Event output depends on the operating point, temporal aggregation, and cooldown. The primary held-out cascade operating point is accompanied by explicit protocol documentation, confidence intervals, and supplementary operating-point analyses. Accordingly, the event-level results from the held-out stream protocol are reported as the primary comparison, whereas the additional split and operating-point analyses are used only as robustness checks.

#### 5.5.2 Personalization through incremental updating

Long-term monitoring requires adaptation to individual physiological baselines and workload changes. The chronological incremental-update analysis used the real nurse-monitoring dataset, the clinical 48D-derived 15D edge-side screening model, and the three held-out nurse streams. To avoid leakage between personalization and evaluation, the protocol was made chronological at the subject level: for each held-out test subject, the first prefix segment was eligible for feedback and model updating, and only the later non-adaptation segment was used for evaluation. Baseline and updated models were compared on the same evaluation segment, but no evaluation window was used to update the model or tune the post-update threshold. A no-update control (adapt_ratio = 0) was also included; in this control, the baseline and incremental branches are identical and the mean ΔF1 is 0.000, confirming that reported gains require actual feedback updates.

A subject-level protocol check confirmed that the three held-out subjects all had zero adaptation/evaluation overlap, and the no-update control remained unchanged across the evaluation segment. With prefix feedback and a fixed post-update threshold, the aggregate micro recall increased from 0.806 to 0.995, but specificity dropped from 0.271 to 0.085. Personalization under this setting therefore acts as a conditional sensitivity-increasing adaptation mechanism with a false-alert trade-off across subjects.

This behavior matches the intended role and risk of personalized incremental learning. Feature distillation lowers edge-side computation and transmission cost, and incremental updating can compensate for mismatch between the static global model and individual time-varying stress responses. However, the no-update control and specificity loss show that the update rule must be calibrated for the target deployment setting. Personalization is therefore a leakage-controlled adaptation experiment with a clear specificity trade-off.

The supplementary results indicate that alert density can be controlled by cascade operating-point selection, while incremental updating can increase sensitivity only with an explicit specificity trade-off.

## 6 Discussion

### 6.1 Implications for nursing innovation and quality improvement

The clinical relevance of the proposed innovation lies in evaluating stress monitoring under nursing-deployment constraints that extend beyond classification accuracy. Continuous nurse-centered monitoring requires timely local response, limited uplink traffic, manageable alert density, and adaptation to longitudinal baseline change. The experiments indicate that compact edge representation, calibrated scoring, cascade confirmation, and personalized updating can be evaluated against these quality-improvement-relevant constraints and showed feasibility within the retrospective setting.

The experimental findings are meaningful for nursing innovation and QI in two respects. On the one hand, the 15-dimensional screening representation retains most of the discriminative utility of the higher-dimensional feature space, which supports the use of compact, sustainable feature construction in resource-constrained wearable environments and aligns with the implementation-science principle of designing innovations that fit real nursing workflows. On the other hand, the cascade alerting structure substantially reduces false alerts and total alert frequency, and its sensitivity–sparsity behavior can be tuned by the gate budget and confirmation threshold. In practical terms, reducing false alerts from 9.68 to 1.88 per hour at the same observed 14/24 event hit count changes the monitoring output from a dense stream that would be difficult to review into a sparser signal that is more compatible with supervisory review, rest prompting, or workload redistribution discussions. This operating-point control is particularly important in nursing settings where excessive alarms are a well-documented source of alert fatigue, cognitive burden, and potential threats to patient safety. Accordingly, the framework may serve as a QI-oriented, nurse-centered early-warning aid for continuous occupational-stress monitoring in future implementation studies.

The system is designed as an assistive monitoring and workflow-awareness approach for nurse-centered occupational-health management. In future workflow evaluations, sustained high-risk states identified by the framework could be examined as triggers for nurse-led QI actions such as closer peer observation during stress-sensitive tasks, workload redistribution, rest prompting, or secondary review of prolonged alert periods. Because the architecture avoids continuous raw-stream upload and relies on compact summaries and lightweight updates, it may be compatible with privacy-sensitive and bandwidth-constrained hospital environments, but practical adoption still requires prospective deployment testing, stakeholder acceptability assessment, and governance rules for how alerts are reviewed and acted upon.

### 6.2 Limitations

The evaluation used WESAD and a public continuous nurse-monitoring dataset, but these data do not cover the full range of departments, wearable platforms, workflow patterns, and institutional practices. Department and shift-type variables were not available in the public metadata used here, preventing stratified analysis by ward type or shift schedule. The nurse-monitoring labels were based on self-reported stress intervals and may contain reporting delay, recall bias, variable event granularity, and mismatch between perceived stress and physiological response. Potential confounders such as medication, caffeine intake, sleep loss, baseline autonomic differences, hydration, and individual health conditions were not available for adjustment. Multi-site validation with richer clinical metadata is needed before the method can be considered broadly transferable.

Latency, communication load, and runtime behavior support edge-side feasibility under the tested deployment-oriented software setting. Actual hospital operation may differ because of sensor dropout, device heterogeneity, wireless instability, battery limits, and shift-specific workflow variation. The present study did not deploy the pipeline on physical wearable hardware and did not measure battery drain or device-level power consumption.

The task formulation is limited to high-risk window discrimination and event-level alerting. The method is intended for continuous monitoring and early warning, with clinical diagnosis remaining outside the task definition. The fully held-out event-level analysis included 24 positive stress events after subject and stress-level filtering under the strict independent stream protocol. This number reflects the available medium/high-stress events in the three held-out nurses under that protocol. Consequently, 10/24 positive test events were not detected by both Single48 and the selected cascade, and the confidence intervals around event recall remained wide. A supplementary all-subject out-of-sample analysis using training-subject out-of-fold predictions plus held-out test-subject predictions increased the positive-event count to 199, and two additional subject-level splits were evaluated; these analyses support the alert-density pattern but are kept secondary to the strict held-out stream test. A missed-event analysis showed that the 10 Single48-missed held-out events had a shorter median duration than the 14 detected events (17.5 vs. 24.0 min) and were concentrated in one held-out subject. Missed events may reflect weak physiological expression, temporal mismatch between self-report and sensor response, movement or sensor artifacts, delayed or anticipatory stress responses, or event contexts that were not captured by EDA, HR/IBI, and temperature features alone. Clinically, wearable alerting can support screening and workflow awareness while self-report, supervisor judgement, and occupational-health assessment remain essential.

Incremental updating improves sensitivity under individual drift and high-stress conditions in the present experiments, but its behavior depends on threshold settings, update intervals, and cohort characteristics. These parameters require recalibration when the method is transferred to new cohorts or deployment protocols. Similarly, the privacy mechanism was evaluated only at the level of summary transmission and raw-stream avoidance; formal privacy guarantees, secure-channel implementation, and attack-resistance evaluation remain future work.

## 7 Conclusions

This work presents NEST, a nurse-centered wearable edge–server framework for occupational-stress monitoring under wearable-device constraints, framed as a contribution to nursing innovation and quality improvement. The retrospective evaluation supports the feasibility of combining compact feature distillation, calibrated local risk scoring, cascade alert confirmation, and personalized incremental updating in a continuous nurse-monitoring pipeline. On WESAD, the 15D representation retained most of the 48D feature set's discriminatory performance. On real continuous nurse-monitoring data, the selected cascade configuration reduced false alerts and total alert frequency in the fully held-out event test while preserving the observed 14/24 event hit count; supplementary 199-event and multi-split analyses indicated that recall and alert burden remain operating-point dependent. Runtime and communication analyses in the edge-equivalent software setting recorded millisecond-level inference, low CPU duty cycle, and reduced uplink traffic, with predictable growth as the number of simulated nodes increased. Chronological incremental updating increased sensitivity when feedback was available, but it did not improve overall F1 and lowered specificity; no-update controls showed no artificial gain. These results support technical feasibility and alert-burden reduction for an assistive QI-oriented monitoring approach. Direct effects on occupational-health outcomes, burnout, nurse retention, workload reduction, or patient safety should be tested in prospective implementation studies. Physical wearable deployment, power measurement, formal privacy evaluation, prospective workflow testing, and broader multi-site implementation studies remain necessary before clinical deployment or claims of transferability.

## Data Availability

The datasets analyzed in this study are publicly available from public repositories and source publications. The Wearable Stress and Affect Detection (WESAD) dataset is available from the UCI Machine Learning Repository at https://archive.ics.uci.edu/dataset/465/wesad+wearable+stress+and+affect+detection and is associated with the dataset DOI https://doi.org/10.24432/C57K5T. The continuous nurse-monitoring dataset, a multimodal sensor dataset for continuous stress detection of nurses in a hospital, is available from Dryad at https://doi.org/10.5061/dryad.5hqbzkh6f. No new human-participant data were collected for this study, and no participant recruitment, intervention, or direct interaction was involved. All data were used in accordance with the conditions of use and source publications of the corresponding public datasets.
